# A polygenic score for schizophrenia predicts glycemic control

**DOI:** 10.1038/s41398-017-0044-z

**Published:** 2017-12-18

**Authors:** Han Cao, Junfang Chen, Andreas Meyer-Lindenberg, Emanuel Schwarz

**Affiliations:** Department of Psychiatry and Psychotherapy, Central Institute of Mental Health, Medical Faculty Mannheim, Heidelberg University, Mannheim, Germany

## Abstract

Schizophrenia is substantially comorbid with type 2 diabetes (T2D), but the molecular basis of this effect is incompletely understood. Here, we show that a cortical schizophrenia expression score predicts glycemic control from pancreatic islet cell expression. We used machine learning to identify a cortical expression signature in 212 schizophrenia patients and controls, which explained ~25% of the illness-associated variance. The algorithm was predicted in expression data from 51 subjects (9 with T2D), explained up to 26.3% of the variance in the glycemic control indicator HbA_1c_ and could significantly differentiate T2D patients from controls. The cross-tissue prediction was driven by processes previously linked to diabetes. Genes contributing to this prediction were involved in the electron transport chain as well as kidney development and support oxidative stress as a molecular process underlying the comorbidity between both conditions. Together, the present results suggest a molecular commonality between schizophrenia and glycemic markers of type 2 diabetes.

## Introduction

Patients with schizophrenia die, on average, about two decades earlier than healthy peers, an excess mortality largely due to somatic illnesses such as type 2 diabetes (T2D)^1^. T2D prevalence is increased 2–3-fold compared with the general population and family history of the illness is more common^2,3^. While T2D can be a consequence of antipsychotic treatment, glycemic alterations have been found in antipsychotic naive subjects, supporting a disease intrinsic molecular comorbidity between the two conditions^4,5^. Although at the genome-wide level schizophrenia and T2D show no genetic correlation^6^, molecular investigations found shared biological alterations in both illnesses. These include elevated levels of insulin and closely related molecules such as IGF, a metabolic profile also found in treated as well as medication-naive patients with schizophrenia^4,7^. On a candidate gene basis, individual risk variants have been implicated in both conditions^8^, supporting shared underlying genetic determinants. At a systems level, mitochondrial dysfunction has been suggested as unifying biological theme underlying T2D and schizophrenia^9,10^. In schizophrenia, increased mitochondrial β oxidation and upregulation of insulin signaling proteins are thought to indicate a state of glucose/energy starvation in the prefrontal cortex that may, in turn, lead to increased oxidative stress^9^. In T2D, abnormal skeletal glucose transport plays a major part in the molecular etiology of insulin-resistant T2D^11^. This defect is thought to arise from fatty acid-induced inhibition of insulin receptor (IRS-1) phosphorylation, potentially due to intramyocellular fatty acid accumulation that may result from abnormal mitochondrial fatty acid oxidation^11^. At the same time, mitochondrial dysfunction in pancreatic β-cells leads to an increase of reactive oxygen species that is thought to underlay the progressive development of β-cell failure, a central part of the T2D pathology^12^. Despite these intriguing data, it remains unclear whether molecular commonality of these two disorders can be demonstrated at a biological systems level.

To address this, we used a machine learning approach to identify a polygenic schizophrenia signature and explored its impact on T2D (Supplementary Fig. S1). The first aim was to identify a signature of genes expressed in the human cortex that could optimally differentiate schizophrenia patients from healthy controls. For this, we used transcriptome-wide cortical expression data from 212 schizophrenia patients and controls. We then predicted this signature in independent pancreatic islet cell expression data from 51 individuals (9 T2D patients). We tested the hypothesis whether the predicted schizophrenia scores were associated with glycated hemoglobin (HbA_1c_) levels, a quantitative readout of glycemic control, where values above 6.5% have been suggested as a diagnostic test for diabetes^13^. The cross-tissue prediction performed here was based on the assumption that (I) schizophrenia is associated with molecular alterations that are at least partially systemic and can be detected in central as well as peripheral tissues and (II) that such alterations are consistent in the direction of their change. We tuned the polygenic model toward schizophrenia relevant biological processes, through pre-selection of genes among gene ontology categories most associated with genetic schizophrenia risk^14^. This also allowed exploration of whether peripheral effects of the schizophrenia signature were masked by those more strongly linked to risk, which may be more brain specific.

## Methods

### Data sets and preprocessing

Transcriptome-wide expression data from four post mortem data sets of schizophrenia patients and controls (GSE53987, GSE21138, GSE35977, and GSE12679) were used to identify a polygenic schizophrenia model in the brain. A data set of pancreatic islet cell expression (GSE38642) was used to test the association of a predicted schizophrenia score with glycemic control. A further data set comprising transcriptome-wide expression data from pancreatic beta cells (GSE25462), acquired from T2D patients and controls using laser capture microdissection, was used for validation of the cross-tissue prediction. Finally, frontal cortex expression data sets from patients with HIV encephalitis (GSE3489) and Alzheimer’s disease (GSE36980) were used as negative controls. Data sets were identified through manual search from the GEO database (freeze date for search February 2017) and details of the data sets can be found in Supplementary Tables S1 and S2.

Out of six identified cortical post mortem expression data sets comprising schizophrenia patients, two (GSE17612 and GSE21935) were excluded due to high average age (70.6 years vs. 44.7 in the remaining four data sets). Data were preprocessed using the robust multi-array average function of the R package affy, performing background correction, log2 transformation, and quantile normalization^15,16^. Multiple reads mapping to the same gene symbol were averaged. To exclude potential outliers, we visually inspected the first two principal components determined for each data set and excluded one patient in GSE53987 and two in GSE21138. Expression data were filtered to contain only genes that overlapped across all discovery studies, resulting in a total gene number of 17,062. To make overall expression levels comparable between brain and pancreatic islet or negative control data, data were quantile normalized again based on the, respectively, overlapping set of measured genes.

### Covariate adjustment and propensity score matching of expression data

To prevent an impact of potential covariates on the ability to derive a polygenic profile from expression data, data sets were first normalized with respect to these variables. Specifically, for each of the schizophrenia brain expression data sets, we determined residuals after regressing expression information from each gene against site, gender, age, and post mortem interval (PMI), as well as the second polynomial of age. If a data set contained more controls than patients, this step was preceded by propensity score matching to identify a 1:1 matched sample based on age, gender, and PMI. This was performed using the R library MatchIt^17^. Pancreatic islet expression data were residualized against the same covariates, except for site. Brain pH was not used as a covariate, since measures were not fully available for all data sets. The pancreatic beta-cell validation data, as well as the Alzheimer’s disease negative control data, were residualized against the same covariates. The former data set was additionally residualized against the first principal component determined from the entire expression data set. This was performed since the authors reporting this study showed the first principal component to capture sample measurement dependent effects on overall variation^18^. Since no metadata were available for the HIV negative control data set, this data set was residualized only against its first principal component.

### Gene selection and comparison of expression levels across tissues

To identify genes to be used for algorithm training, we selected those part of gene ontology categories that have previously been associated with schizophrenia, based on genome-wide functional analysis of GWAS data^14,19^. The selection was performed among the genes linked to schizophrenia (single disorder analysis) and focused on gene ontology only, since of the 4949 gene sets that were investigated by the PGC and annotated by five databases (GO, KEGG, Panther, Reactome, TargetScan), 4550 were from the gene ontology^14^. Among these, we selected the top 200 categories to obtain a likely over-inclusive list of genes. Subsequently, we explored the impact of removing genes in the most schizophrenia-associated categories. For this, we removed genes in the top 0, 20, 40, 60, 80 and 100 categories and corrected the resulting *P*-values for the family-wise error rate (FWER) according to the method of Bonferroni.

To compare expression levels between brain and pancreatic tissue, we used RNA-Seq data from human tissue samples from the genotype-tissue expression (GTEx) project^20^, available from the Expression Atlas^21^. The conventional 0.5 FPKM (fragments per kilobase of exon model per million reads mapped) was used as expression cut-off.

### Machine learning strategy

The machine learning strategy employed here was aimed at deriving a signature from a data set with correlated variables and a variable number that greatly exceeded the number of samples. Therefore, we utilized a strategy devised for this purpose that consisted of the following steps^22^: (I) determine the top *n* genes associated with case–control status. We used linear correlation for this purpose and chose *n* to be the sample number divided by 10. This selection was made since expression data were expected to show sufficient effect sizes such that accurate classifiers can be derived from a relatively small number of good predictors^23^. (II) Build a binomial model for case–control prediction via penalized maximum likelihood to further reduce the variable number to the most important predictors. Five-fold cross-validation was used to identify the optimal lambda value that determines the number of predictors. (III) Residualize the outcome variable against these predictors using a binomial model. (IV) Repeat the procedure from step (I) *m* times. We chose *m* = 2, such that the total number of predictors remained below 50^24^. (V) Using these predictors, build a smoothly clipped absolute deviation-penalized binomial regression model for prediction of diagnosis. Again, cross-validation was used to identify the optimal lambda value. (VI) Predict the final model in the pancreatic islet data.

The benefit of this method is its ability to efficiently select important predictors and cope with potential correlation among them. Compared to its original implementation, we made one further adaptation that can substantially improve classifier performance. Instead of training the classifier on the entire data set, we repeatedly trained it on subsets of the training data and averaged the prediction outcome. This method is also known as bootstrap aggregation (“bagging”) and integral part of other powerful machine learning tools, such as random forests^25^. Since our training data consisted of four different brain expression data sets, we randomly selected two-thirds of patient and control samples from each data set individually and combined them to form the training data. For the brain expression data, classification performance was tested on the samples not used for training, averaged over 1000 repetitions of the procedure. Classification accuracy was measured using non-parametric correlation between predicted and actual glycemic control values and using Nagelkerke’s *R*^2^ from logistic regression for classification of schizophrenia.

To demonstrate the specificity of associations between the schizophrenia polygenic model and glycemic control, the analysis was repeated 1000 times, starting with permuted diagnosis information in the brain expression data. We determined an empirical *P*-value as the frequency of explained variance estimates at least as high as the one observed for the original diagnostic information.

### Weighted gene co-expression network analysis

Weighted gene co-expression network analysis (WGCNA) was performed using the R package WGCNA^26^, using only control subjects of the residualized cortical schizophrenia expression data. This analysis was performed on the combined data (rather than consensus analysis across individual dat asets) since, due to the small sizes of individual data sets, removal of associations with potential confounders may be more robust in the combined data. We identified a soft-threshold (the lowest *β* value to lead to an *R*^2^ of >0.80^27^, in the present analysis *β* = 4) to fit a scale-free topology to the network. The weighted adjacency matrix was then transformed into topological overlap (TOM) and hierarchical clustering (using flashClust^28^) was used to identify modules from the TOM dissimilarity matrix (1-TOM). We used a relatively small minimum module size of 10 to allow identification of gene sets with low gene numbers that may have high cross-tissue predictiveness. Although small module sizes may lead to biologically less plausible modules, this risk was minimized in the present study by performing WGCNA on a preselected set of genes from few gene ontology categories.

## Results

### Identification of a cortical schizophrenia signature

**Fig. 1:**
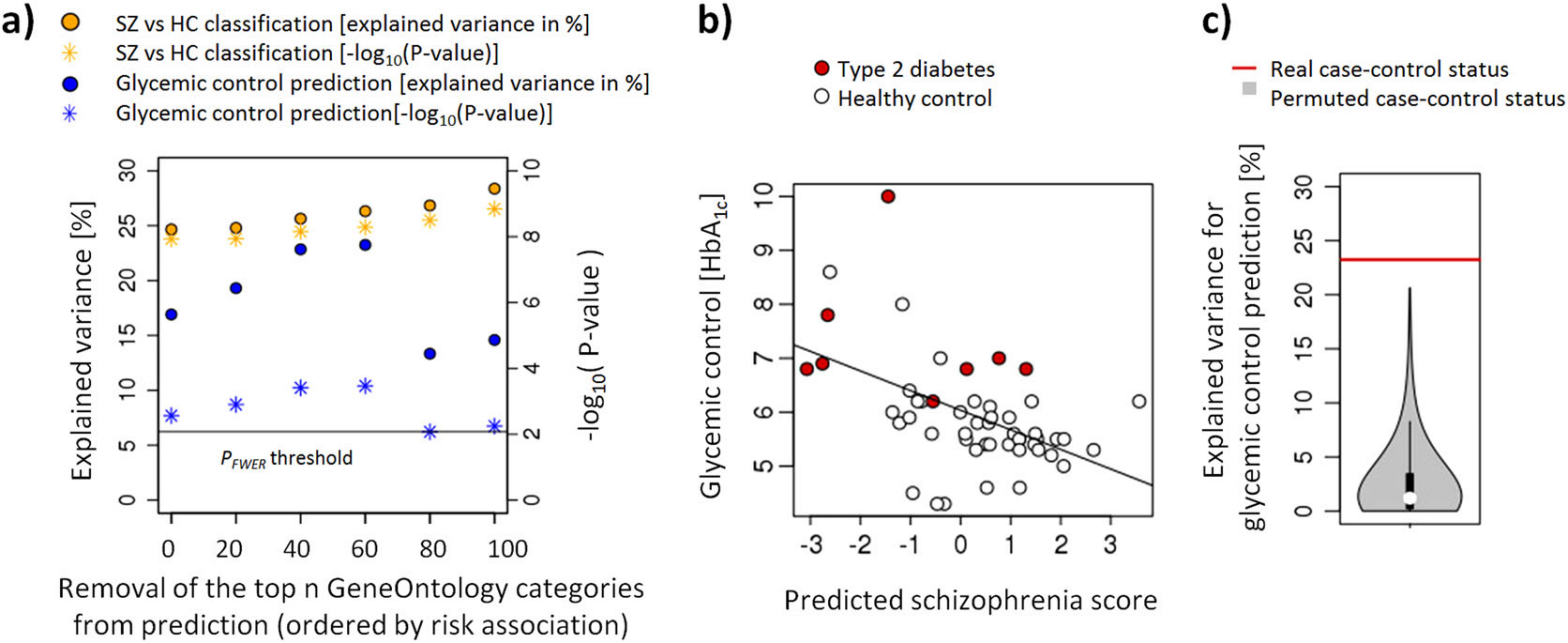
Schizophrenia polygenic model predicts glycemic control **a** Accuracy for HbA_1c_ and case–control status prediction. The former was more accurate when the 60 ontological categories most associated genetic schizophrenia risk were excluded. **b** Association between the schizophrenia score and glycemic control. **c** Explained variance in glycemic control prediction for permuted and real schizophrenia diagnosis. SZ schizophrenia, HC healthy control

Machine learning was used to identify a cortical gene expression signature in 212 schizophrenia patients and controls. The model was built using genes within the 200 ontological categories that have previously been reported to be most strongly associated with genetic schizophrenia risk. Of these, 170 contained at least one gene measured across all investigated data sets (median number of genes was 14). Fig. 1a shows the polygenic model performance in schizophrenia brain expression data. The explained variance for classification of schizophrenia had a median of 25% with a median *P*-value of 6.1×10^−9^ and this did not depend on genes within ontological categories most associated with genetic schizophrenia risk. Specifically, performance was similar when genes part of the top 20, 40, 60, 80, and 100 ontological categories were removed prior to machine learning analysis.

### Association of predicted schizophrenia scores with glycemic control

Fig. 1a further shows that HbA_1c_ levels could be predicted for all investigated gene sets below the FWER-corrected *P*-value threshold of 0.008. Notably, prediction performance peaked after removing genes within the top 60 ontological categories, suggesting that gene sets with the strongest genetic schizophrenia association were adversely affecting the prediction of glycemic control. At this threshold, the polygenic schizophrenia model explained 26.3% of variance in the glycemic control index (rho = −0.48, *P* = 0.0003, Fig. 1b) and differentiated T2D patients from healthy controls (*P = *0.021, Wilcoxon test). Permuting diagnosis demonstrated the specificity of the findings for the real case–control status (*P < *0.001, Fig. 1c, the median explained variance of schizophrenia classification during permutation was 0.8%). To explore the sensitivity of the results to the arbitrarily selected cut-off of 200 GO categories, the analysis was repeated for the 300 and 500 categories with the strongest genetic associations with schizophrenia. In both analyses, predicted schizophrenia scores were significantly associated with HbA_1c_ levels (rho = -0.34, *P* = 0.015 and rho = −0.32, *P* = 0.023, respectively).

Next, we explored whether the bottom 200 ontological categories regarding genetic schizophrenia risk could also predict glycemic control. This was not the case with an explained variance of 3.9% (*P = *0.17), even though the schizophrenia-control differentiation was not strongly affected (26% variance explained, *P = *1.0×10^−8^).

### Identification of genes underlying cross-tissue prediction

**Fig. 2:**
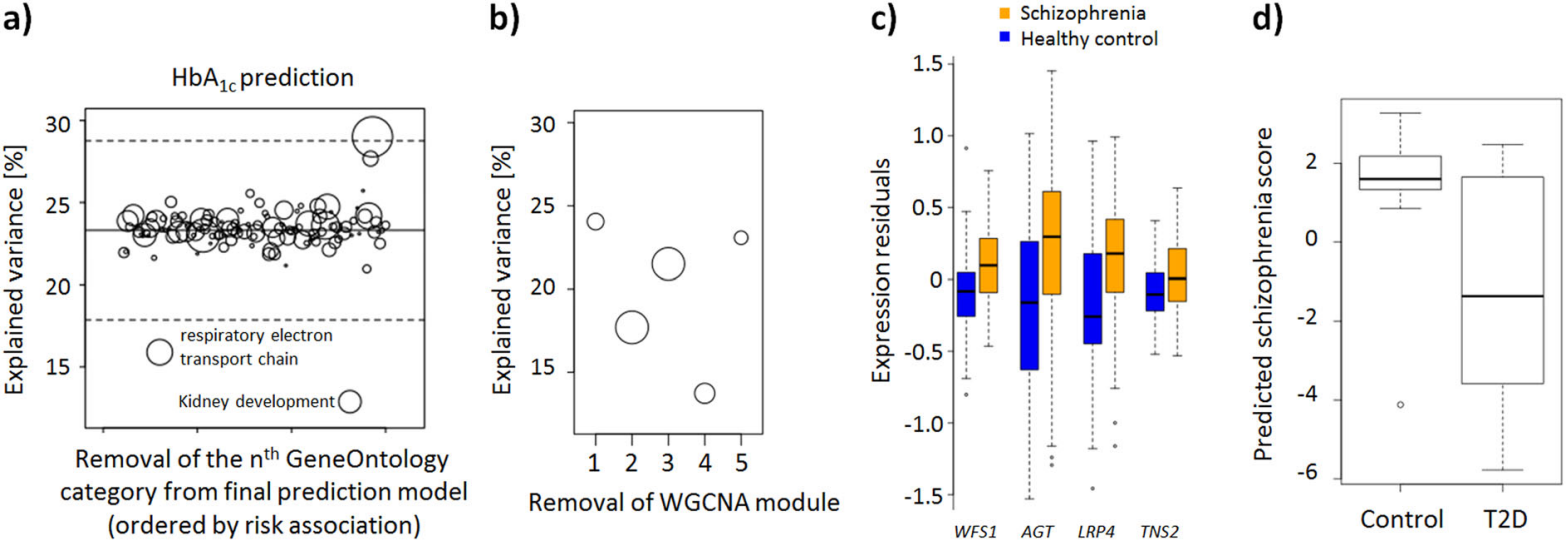
Importance for individual ontological categories and genes for HbA1c prediction **a** Explained variance after excluding individual categories from the polygenic model, starting with the best model from Fig. 1. Circle radius is proportional to the number of genes part of the respective category. The solid line shows the mean explained variance, the dotted lines the 3.53 SD interval (FWER corrected at *P* = 0.05). **b** Explained variance after excluding individual modules from WGCNA of genes in the two categories shown in a. **c** Boxplots of the four genes in WGCNA module four most associated with schizophrenia. **d** Validation cohort: difference of predicted schizophrenia score between pancreatic beta cells of T2D patients and controls

To identify ontological categories important for the cross-tissue prediction, we successively removed genes in each of the 140 remaining ontological categories from the analysis. Figure 2a shows the two most important categories were “kidney development” (GO:0001822) and “respiratory electron transport chain” (GO:0022904). WGCNA identified five gene modules among the two categories with one module of 22 genes contributing most to the decrease in explained variance (Fig. 2b). Among these, the four genes most strongly altered in schizophrenia were *WFS1* (*P* = 1.8×10^−7^), angiotensinogen (*AGT*, *P* = 1.3×10^−6^), *LRP4* (*P* = 2.7×10^−6^), and *TNS2* (*P* = 9.3×10^−4^), all of which were increased in schizophrenia (Fig. 2c; Supplementary Table S3).

GTEx RNA-seq data were used to compare expression levels of genes part of the cortical signature between brain and pancreatic tissue. This showed that 78% of the 2897 genes part of the 200 (170 represented with at least 1 gene) ontological categories most associated with schizophrenia risk were expressed in the dorsolateral prefrontal cortex (BA9) at a cut-off of 0.5 FPKM. For pancreatic tissue, this was the case for 70% of such genes. For genes part of the two identified ontological categories, the corresponding percentages were 82% (BA9) and 80% (pancreas), respectively. Supplementary Figure S2 shows a comparison of expression levels of these genes between tissue types.

### Validation and negative control

To validate the cross-tissue prediction based on genes in the identified ontological categories, we used an additional, independent pancreatic expression data set, comprising 10 T2D patients and 10 healthy controls. Schizophrenia scores were predicted using all 162 genes part of the ontological categories “kidney development” and “respiratory electron transport chain” and which were shared between the brain and pancreatic expression data sets. The algorithm derived from these genes explained 23% of the variance (*P* = 2.7×10^−8^) for classification of schizophrenia. Prediction in pancreatic beta cells showed that T2D patients had significantly lower predicted schizophrenia scores compared to controls (*P* = 0.008, corrected for the influence of age and sex, Fig. 2d), consistent with findings from the pancreatic expression data set described above.

Two analyses were performed as a negative control. First, schizophrenia scores were predicted in a frontal cortex expression data set of subjects with HIV encephalitis (*n* = 16) and controls (*n* = 12) to support the specificity of the schizophrenia signature for prediction in comparable brain tissue samples. As above, the prediction was based on genes part of the two identified ontological categories and which overlapped with the schizophrenia data set (127 genes). This showed that predicted scores did not differ between HIV encephalitis patients and controls (*P *= 0.54).

Second, scores were predicted in frontal cortex expression data of subjects with Alzheimer’s disease (*n* = 15) and healthy controls (*n* = 18) using 162 overlapping genes of the two ontological categories. Also here, predicted schizophrenia scores were not associated with case–control status (*P *= 0.64, corrected for the influence of age and sex).

## Discussion

Here, we show that a glycemic marker of T2D can be predicted using a schizophrenia brain expression score. This finding supports the presence of a systemic molecular commonality between schizophrenia and T2D. This is in agreement with studies identifying consistent alterations of individual molecules in the periphery and central nervous system of schizophrenia patients (e.g.,^29^). The association between the score and HbA_1c_ levels was negative, meaning that subjects with a pancreatic islet profile more similar to that of schizophrenia patients had lower HbA_1c_ levels. Consistent with this, T2D patients in this cohort as well as in an independent validation cohort had significantly lower schizophrenia scores compared to controls. There are several potential explanations for this finding. First, brain changes underlying the score may be of protective nature or affected by compensatory mechanisms. It is conceivable that such mechanisms are influenced by illness duration and the effects of antipsychotic medication. For example, a shared risk process may initially lead to comparable expression profiles. During later illness stages of schizophrenia, this process may be over-compensated, leading to an inversion of associations with HbA_1c_ levels. This hypothesis could be examined by exploring potential interactions between the predicted schizophrenia score, pancreatic HbA_1c_ levels, and illness duration. Such analysis would be particularly informative if schizophrenia patients would show similar expression profiles in peripheral cells. This would allow testing of samples from living and potentially untreated patients. A second possibility may be a shared risk mechanism that results in opposite expression effects in different tissues. To explore this, it would be interesting to investigate genetic risk overlap between schizophrenia and T2D in the identified processes. Potential joint risk signatures could then be examined regarding their tissue-specific, quantitative effects on expression.

Interestingly, prediction of HbA_1c_ levels improved when gene sets with the highest genetic schizophrenia association were excluded. This implies that these genes were good predictors for schizophrenia with little relevance for HbA_1c_ prediction. Therefore, their removal eliminated noise from the HbA_1c_ prediction and led to improved performance. We further observed that genes relevant for the cross-tissue prediction clustered within the ontological categories more strongly linked to genetic schizophrenia risk. This may point toward genetic comorbidity effects within the identified signature but requires further elaboration.

We used data from subjects with Alzheimer’s disease and HIV encephalitis as a negative control for the present study. Interestingly, the pathology of both conditions has been linked to oxidative stress, supporting the specificity of the identified cortical signature for schizophrenia^30,31^.The two ontological categories contributing most to the prediction of glycemic control were “kidney development” and “respiratory electron transport chain”. While HbA_1c_ levels are significantly predictive of chronic kidney disease^32^ and T2D is strongly associated with reduced kidney function and risk of kidney failure^33,34^, accumulating evidence suggests that the kidneys are directly involved in glucose homeostasis and insulin metabolism^35^. For example, insulin resistance is more frequent in patients with acute kidney injury^36^ and glucose uptake is decreased in uremia^37^. Renal glucose production and removal account for 25% and 20% of systemic production and removal, respectively^38^, supporting the kidneys’ central role in glucose homeostasis. Together, these reports suggest that kidneys play an important role in inducing abnormal glucose homeostasis and are also a target of the downstream consequences in the form of renal injury^35^.

The important predictive role of genes associated with the “respiratory electron transport chain” further supports mitochondrial dysfunction and oxidative stress as the unifying theme underlying the comorbidity between schizophrenia and T2D^9,10^. The respiratory electron transport chain is a key part for oxidative phosphorylation of glucose, which has been identified as the most significantly downregulated pathway in schizophrenia post mortem brains^9^. It is also known to be deficient in skeletal muscle mitochondria of T2D patients^39^ and oxidative stress due to aberrant oxidative phosphorylation plays an important role in the development of diabetic nephropathy^40^. The predictive importance of oxidative phosphorylation and kidney-related genes identified here further supports their joint relevance for glucose homeostasis both in schizophrenia as well as T2D.

The most strongly implicated gene, *WFS1*, encodes the transmembrane protein Wolframin. Mutations in this gene can lead to Wolfram syndrome that presents with insulin-dependent diabetes mellitus^41^. Consistent with this, *WFS1* variants have frequently been reported to contribute to T2D risk (e.g.,^42–44^). Two SNPs (rs10010131 and rs6446482) with intronic locations in the *WFS1* gene are reproducibly associated with a protective effect on risk for T2D. The gene is characterized by a strong LD pattern and, albeit not consistently, evidence for further genetic risk associations has been reported. Meta-analysis investigating rs10010131 and rs734312 has confirmed significant protective effects for the minor alleles of both variants^45^, although the latter SNP may not be functionally relevant^42^. The risk allele of rs10010131 is further predictive of future T2D, progression from normal glucose tolerance to T2D^46^, as well as insulin secretion^47^. This allele has also been found to interact with a variant in hepatocyte nuclear factor 4 alpha (*HNF4A*) in an Ashkenazi Jewish Population, suggesting a potential gene–gene interaction effect on T2D risk^48^.

Interest in WFS1’s role in schizophrenia stems from observations that mutation carriers show an increased likelihood for psychiatric hospitalization^49–51^. Several subsequent genetic studies have investigated potential associations with schizophrenia risk, but these findings have thus far not supported a significant role^52^. For example, the T2D-associated variant rs10010131 has not been found associated with schizophrenia in a Danish cohort of 410 patients and 820 controls^53^. Similarly, WFS1 is not harbored by the well-established 108 genetic loci associated with schizophrenia risk^54^.

We further identified angiotensinogen as a gene important for the cross-tissue prediction in the present study. Angiotensinogen is a precursor of angiotensin I, a central part of the renin-angiotensin system (RAS), an important regulator of glucose homeostasis^55^. In T2D, tissue RAS is activated leading to increased oxidative stress and progressive renal pathology [56]. While an angiotensinogen variant (rs699) that has previously been linked to hypertension, has not been found associated with T2D, such association has been identified for a variant in another gene central to the RAS (aldosterone synthase)^57^.

In schizophrenia, research into the RAS has focused on angiotensin converting enzyme (ACE), which converts angiotensin I into angiotensin II but genetic association analyses have thus far been inconclusive^58^. The etiological role of the schizophrenia candidate gene *ACE* was initially thought to relate to its involvement in dopamine metabolism^59,60^. More recently, an *ACE* polymorphism has been shown to be associated with plasma glucose concentrations in chronic schizophrenia patients^61^. Together with the present findings, this may support the involvement of the RAS system in the comorbidity between schizophrenia and T2D, potentially due to their role in regulating glucose homeostasis.

A further gene important for cross-tissue prediction identified here is the LDL receptor-related protein 4 (LRP4). It belongs to the family of lipoprotein receptor-related proteins (LRPs) and antagonizes LRP6-mediated activation of canonical Wnt signaling. Mutations in this gene impact on such signaling and are associated with kidney anomalies in Cenani–Lenz Syndrome^62^. LRP5, a co-receptor of LRP6, has a central role in glucose-induced insulin secretion from pancreatic islets and for maintenance of normal cholesterol metabolism^63^. Polymorphisms in LRP5 are associated with obesity^64^ and variants in several other Wnt signaling genes have been linked to the development of T2D, most prominently in *TCF7L2*^65^. Notably, Wnt signaling plays an important role in kidney development and regulates the expression of hormones essential for glucose homeostasis^66^.

Numerous studies have explored the role of Wnt signaling in schizophrenia, mainly due to its involvement in neuronal development^67^. Interestingly, a T2D risk variant in *TCF7L2* has also been found to increase the risk for schizophrenia and this allele is associated with increased expression in pancreatic beta cells^53^. Further studies should explore the relationship between variation in Wnt signaling-related genes and glucose homeostasis as a potential factor underlying the comorbidity of schizophrenia and T2D. For example, another gene identified here, *TNS2 (TENC1)*, has previously been found to interact with schizophrenia risk gene *DISC1*^68^ and is involved in the regulation of Akt, an important modulator of Wnt signaling^69,70^.

The most significant limitation of the present study is that several confounding effects on the brain expression signature cannot be excluded. Although schizophrenia confers a significant endogenous risk for T2D, this risk is further increased by antipsychotic treatment^71^. Such medication effects may have influenced the identified cortical signature. Additional confounders include nicotine, alcohol, or drug use as well as the mode of death (i.e., suicide). Therefore, the molecular commonality identified in the present study should be interpreted as a state-dependent commonality between schizophrenia and T2D. Further studies are warranted to explore potential associations between this molecular overlap and factors underlying the genetic comorbidity between the conditions.

## Conclusion

This study supports the presence of a molecular brain signature of schizophrenia that is associated with a glycemic marker of T2D in the periphery. These findings may help to elucidate the biological basis of comorbidity between the two illnesses. They may also aid in uncovering processes underlying the impact of antipsychotic treatment on T2D risk and highlight potential molecular targets for treatment of this clinically relevant comorbidity.

## Electronic supplementary material

Supplementary Figures

Supplementary Tables
