# Supplementary material for: A polygenic score for schizophrenia predicts glycemic control
Source: Transl Psychiatry. 2017 Dec 18;7:1295. doi: 10.1038/s41398-017-0044-z (PMC5802590; doi:10.1038/s41398-017-0044-z)
Supplement: Supplementary file 1 — Supplementary Figures [file 41398_2017_44_MOESM1_ESM.docx]

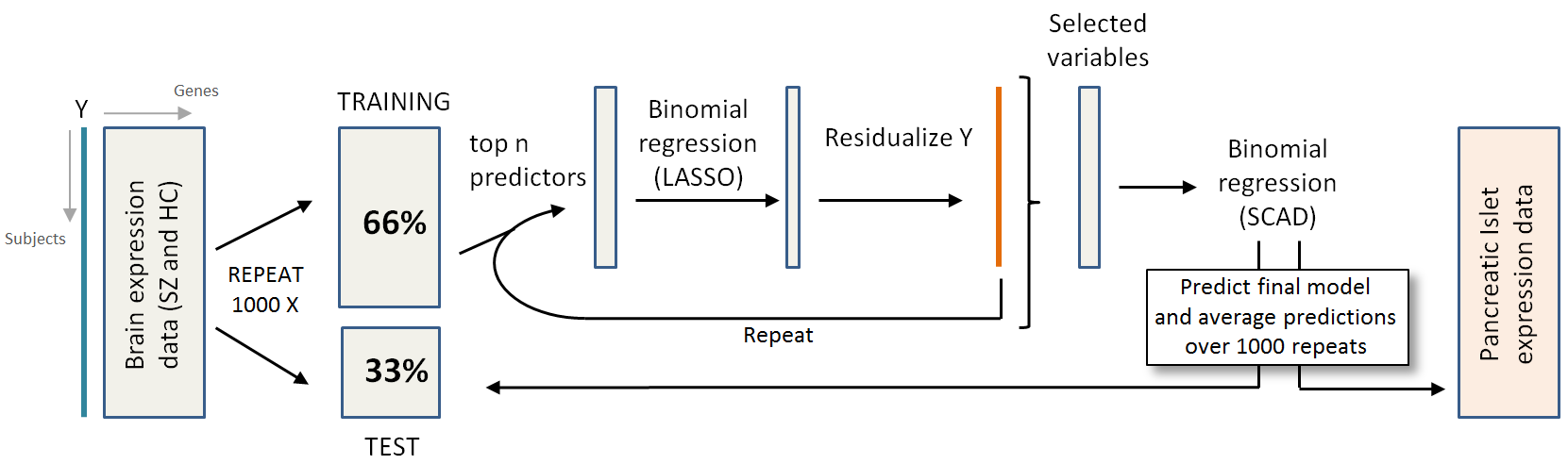


**Figure S1.** **Schematic overview of machine learning work-flow.** First, the top n schizophrenia associated genes are identified in cortical post-mortem expression data. Regularized binomial regression further reduces predictor numbers and uses these to residualize Y. This is repeated and the final variable set used to predict (I) case-control status in samples not used for training and (II) pancreatic islet expression data. The procedure is repeated 1000 times and predictions are averaged. The pancreatic prediction is then used to determine associations with glycemic control. SZ: Schizophrenia; HC: Healthy control.


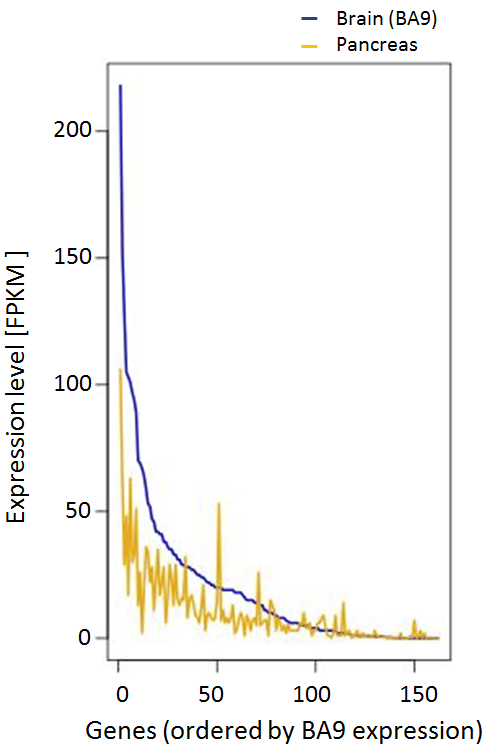


**Figure S2.** **Comparison of expression levels in brain (BA9) and pancreatic tissue.** Expression levels quantified as FPKM (fragments per kilobase of exon model per million reads mapped) for 162 genes part of the ontological categories “kidney development” and “respiratory electron transport chain”. Genes were ordered based on brain expression.
