# Supplementary material for: A polygenic score for schizophrenia predicts glycemic control
Source: Transl Psychiatry. 2017 Dec 18;7:1295. doi: 10.1038/s41398-017-0044-z (PMC5802590; doi:10.1038/s41398-017-0044-z)
Supplement: Supplementary file 2 — Supplementary Tables [file 41398_2017_44_MOESM2_ESM.docx]

**Table S1. Demographics of subjects used for expression analysis.** Values are shown as mean ± sd.

|  | Schizophrenia | | | |  | Diabetes |
| --- | --- | --- | --- | --- | --- | --- |
|  | GSE53987 | GSE21138 | GSE35977 | GSE12679 |  | GSE38642 |
| Reference | ^1^ | ^2^ | ^3^ | ^4^ |  | ^5^ |
| Tissue | Fontal cortex (BA46) | Fontal cortex (BA46) | Parietal cortex | Dorsolateral prefrontal cortex (BA9)* |  | Pancreatic islets |
| n patients | 14 | 28 | 51 | 16 |  | 9 (T2D) |
| n controls | 14 | 28 | 50 | 11 |  | 42 |
| age patients | 46 ± 9 | 44 ± 17 | 43 ± 10 | 44 ± 8 |  | 56 ± 14 |
| age controls | 46 ± 10 | 45 ± 16 | 46 ± 9 | 41 ± 8 |  | 55 ± 10 |
| sex patients (m/f) | 7/7 | 23/5 | 37/14 | 11/5 |  | 4/4 |
| sex controls (m/f) | 8/6 | 23/5 | 35/15 | 8/3 |  | 26/17 |
| PMI patient | 19 ± 7 | 38 ± 12 | 31 ± 16 | 29 ± 9 |  | 29 ± 5 |
| PMI controls | 20 ± 3 | 40 ± 14 | 27 ± 12 | 24 ± 16 |  | 26 ± 3 |
| brain pH patients | 6.5 ± 0.4 | 6.3 ± 0.2 | 6.4 ± 0.3 | - |  | - |
| brain pH controls | 6.6 ± 0.2 | 6.3 ± 0.2 | 6.5 ± 0.3 | - |  | - |

*The dataset consists of endothelial cell and neuronal samples from an overlapping population

**Table S2. Demographics of validation and negative control data.** Values are shown as mean ± sd.

|  | Diabetes | HIV encephalitis | Alzheimer‘s |
| --- | --- | --- | --- |
|  | GSE25462 | GSE3489 | GSE36980 |
| Reference | ^6^ | ^7^ | ^8^ |
| Tissue | Pancreatic beta-cells | Fontal cortex (BA46) | Fontal cortex |
| n patients | 10 | 16 | 15 |
| n controls | 10 | 12 | 18 |
| age patients | 67.3 ± 6.9 | - | 92.9 ± 6.3 |
| age controls | 60.3 ± 4.9 | - | 80.1 ± 8.9 |
| sex patients (m/f) | 7/3 | - | 7/8 |
| sex controls (m/f) | 6/4 | - | 9/9 |
| PMI patient | - | - | - |
| PMI controls | - | - | - |
| brain pH patients | - | - | - |
| brain pH controls | - | - | - |

**Table S3. Expression differences between schizophrenia and control in WGCNA module 4.** All genes with nominal *P* < 0.05 are shown.

| Gene | Name | P-value | Increased in schizophrenia |
| --- | --- | --- | --- |
| WFS1 | Wolframin | 1.8·10^-7^ | Yes |
| AGT | Angiotensinogen | 1.3·10^-6^ | Yes |
| LRP4 | LDL Receptor Related Protein 4 | 2.7·10^-6^ | Yes |
| TNS2 | Tensin-2 | 9.3·10^-4^ | Yes |
| TIPARP | TCDD-inducible poly [ADP-ribose] polymerase | 1.4·10^-3^ | Yes |
| ALDH9A1 | Aldehyde dehydrogenase 9 family member A1 | 6.0·10^-3^ | Yes |
| ASS1 | Argininosuccinate synthase 1 | 1.5·10^-2^ | Yes |
| SALL1 | Spalt Like Transcription Factor 1 | 3.3·10^-2^ | Yes |

**References**

1. Lanz TA, Joshi JJ, Reinhart V, Johnson K, Grantham LE, 2nd, Volfson D. STEP levels are unchanged in pre-frontal cortex and associative striatum in post-mortem human brain samples from subjects with schizophrenia, bipolar disorder and major depressive disorder. *PLoS One* 2015; **10**(3)**:** e0121744.

2. Narayan S, Tang B, Head SR, Gilmartin TJ, Sutcliffe JG, Dean B *et al.* Molecular profiles of schizophrenia in the CNS at different stages of illness. *Brain Res* 2008; **1239:** 235-248.

3. Chen C, Cheng L, Grennan K, Pibiri F, Zhang C, Badner JA *et al.* Two gene co-expression modules differentiate psychotics and controls. *Mol Psychiatry* 2013; **18**(12)**:** 1308-1314.

4. Harris LW, Wayland M, Lan M, Ryan M, Giger T, Lockstone H *et al.* The cerebral microvasculature in schizophrenia: a laser capture microdissection study. *PLoS One* 2008; **3**(12)**:** e3964.

5. Taneera J, Lang S, Sharma A, Fadista J, Zhou Y, Ahlqvist E *et al.* A systems genetics approach identifies genes and pathways for type 2 diabetes in human islets. *Cell Metab* 2012; **16**(1)**:** 122-134.

6. Marselli L, Thorne J, Dahiya S, Sgroi DC, Sharma A, Bonner-Weir S *et al.* Gene expression profiles of Beta-cell enriched tissue obtained by laser capture microdissection from subjects with type 2 diabetes. *PLoS One* 2010; **5**(7)**:** e11499.

7. Masliah E, Roberts ES, Langford D, Everall I, Crews L, Adame A *et al.* Patterns of gene dysregulation in the frontal cortex of patients with HIV encephalitis. *J Neuroimmunol* 2004; **157**(1-2)**:** 163-175.

8. Hokama M, Oka S, Leon J, Ninomiya T, Honda H, Sasaki K *et al.* Altered expression of diabetes-related genes in Alzheimer's disease brains: the Hisayama study. *Cereb Cortex* 2014; **24**(9)**:** 2476-2488.
